# Beer, wine and lifestyle: a cross-sectional study of the Belgian military population

**DOI:** 10.1186/s40779-015-0066-x

**Published:** 2015-12-14

**Authors:** Patrick Mullie, Peter Clarys

**Affiliations:** Faculty of Physical Education and Physiotherapy, Laboratory for Human Biometrics and Biomechanics, Vrije Universiteit Brussel, Pleinstraat 2, B-1050 Brussels, Belgium; Unit of Epidemiology and Biostatistics, Queen Astrid Military Hospital, Bruynstraat 1, B-1120 Brussels, Belgium; Erasmus University College, Laarbeeklaan 121, B-1120 Brussels, Belgium

**Keywords:** Beer, Wine, Dietary pattern, Nutritional assessment, Health behavior, Males

## Abstract

**Background:**

A reduction in mortality associated with wine drinking compared to beer drinking has been suggested in the past. A recent meta-analysis could not confirm the observed differential effect. Other characteristics not related to specific components of beer and wine must play a role in the relationship between wine and mortality, thereby explaining the differential protective results.

**Methods:**

A military population was selected to investigate the lifestyle differences between beer and wine drinkers. A food-frequency questionnaire was used to register alcohol and food consumption, together with questionnaires for health-related and lifestyle characteristics. Three dietary patterns were characterized by the Healthy Eating Index 2010, the Mediterranean Diet Score and a pattern obtained by principal component analysis.

**Results:**

In the multivariate analysis, beer consumption decreased with increasing age, military rank, physical activity and dietary pattern scores. Beer consumption increased with total energy intake and with smoking.

**Conclusions:**

Wine consumption was associated with a healthier lifestyle compared with beer consumption. Those differences must be taken into account when relating types of alcoholic beverage consumption with health-related outcomes.

## Background

In the past, it has been suggested that a reduction in mortality may be related to the type of alcoholic beverages consumed. Studies found lower mortality rates associated with wine drinking [1]. The relationship between wine and mortality has been described as a J-shaped curve, with a beneficial effect occurring from low daily wine consumption [2]. In their meta-analysis, Costanzo et al. were the first to establish the same J-shaped curve for beer as for wine [2]. A maximal protection is, on average, 31 % (95 % CI: 19–42 %) and is observed at 21 g/day of wine. Similarly, a J-shaped relationship was apparent for beer, producing a maximal protection of 42 % (95 % CI: 19–58 %) at 43 g/day of beer [2]. This reduced risk may be due to an increased high-density lipoprotein (HDL) cholesterol fraction and/or by the antithrombotic effect of alcoholic beverages [2].

The early observed differential effect of wine compared to beer on mortality would indicate that components other than alcohol play a role in explaining the mortality differences associated with beer and wine. The antithrombotic and anti-inflammatory effects of polyphenolic substances in wine have been proposed as an alternative mechanism [1]. However, recent research contradicted the specific effect of polyphenolic substances on mortality [3]. Thus, other characteristics not related to the specific components of beer and wine must play a role in the relationship between wine and mortality, thereby explaining the differential protective results of wine.

Few studies have been conducted relating the type of alcoholic beverage to dietary patterns. Dietary pattern analysis, based on the concept that the combination of foods eaten are as important as a reductive methodology characterized by a single food or nutrient analysis, emerged more than 10 years ago as an alternative approach for studying the relationship between nutrition and disease [4]. Two major methods are used to reduce complex dietary data: a hypothesis-oriented approach using prior information to stratify a dietary pattern and a statistical approach using study-specific data to rank individuals, i.e., principal component analysis or reduced rank regression models [4].

The Healthy Eating Index 2010 and the Mediterranean Diet Score are two international and frequently used hypothesis-oriented approaches. The Healthy Eating Index 2010 represents the degree to which a dietary pattern conforms to official guidelines summarized in the United States Department of Agriculture Food Guide Pyramid [5]. The Mediterranean Diet Score, according to the Mediterranean dietary pattern, has received much attention due to the associated reduction in mortality [6]. A statistical approach can also be used to derive a dietary pattern through principal components analysis.

The aim of this study was to investigate the relationship between beer and wine consumption and dietary patterns. In the present study, three dietary patterns were characterized by the Healthy Eating Index 2010, the Mediterranean Diet Score and a pattern obtained by principal component analysis.

## Methods

After stratification for military rank and age, 5000 men were selected as a random representative sample of the Belgian army structure totaling 33,053 men. The selection consisted of 598 officers, 2103 non-commissioned officers and 2299 soldiers. A semi-quantitative food frequency questionnaire with 150 food items was mailed to the participants to record consumption during the last year. The following categories of consumption frequency were used: never, one to three times a month, once a week, two to four times a week, five to six times a week, once a day, two to three times a day, four to six times a day and more than six times a day. Portion sizes were predefined using familiar measuring devices (such as teaspoons, glasses, and cups). The validity of the questionnaire was tested on a representative sample of 100 men in the cross-sectional study [7].

Beer and wine consumption was totaled for each participant in ml/day. A glass of beer was converted to 250 ml, and a glass of wine was converted to 100 ml. Beer consumption was the sum of all types of beer consumed by an individual. Wine consumption was defined as the sum of red, white and rosé wine consumed by an individual. For the analyses, beer consumers were defined as participants for whom 60 % or more of their total alcohol consumption was beer and wine consumers were defined as participants for whom 60 % or more of their total alcohol consumption was wine.

A second questionnaire about physical activity based on validated translations of the International Physical Activity Questionnaire (IPAQ) [8] was sent to the participants. The IPAQ short form is an instrument designed for population surveillance of physical activity among adults. IPAQ registers physical activity across different domains such as leisure time activity, domestic and gardening activity, and work- and transport-related activity.

A third questionnaire was used to register health-related and lifestyle characteristics. This questionnaire was a more general questionnaire about smoking, age, weight, and height. This questionnaire was used in previous research [9].

Participation was on a voluntary basis and without incentives. This study conformed to the guidelines of the Declaration of Helsinki, and all procedures involving human subjects were approved by the Bioethical Committee of the University of Leuven. Written informed consent was obtained from all subjects.

### Statistical analysis

The differences in the proportion of officers, non-commissioned officers and soldiers between responders and non-responders were tested using chi-square tests for categorical data and unpaired *t*-tests for continuous data.

The Healthy Eating Index 2010 and the Mediterranean Diet Score were computed as described earlier [5, 6]. The possible scores for the Healthy Eating Index ranged from 0 to 100 and for the Mediterranean Diet Score from 0 to 9, with a higher score indicating a healthier pattern. Principal component analysis was applied to the semi-quantitative food frequency questionnaire data. First, 150 food items were classified into 34 predefined food groups with similar nutrient profiles, according to Hu et al. [4]. Principal components analysis was used to derive dietary patterns based on the 34 food groups. Varimax transformation was performed to identify uncorrelated factors in order to increase interpretability. Components with eigenvalues of more than 1.5, interpretability of the factors and the Scree plot were used to determine the number of selected factors. The eigenvalues of the factors dropped after the second factor (from 2.44 to 1.77) and after the third factor (from 1.77 to 1.44). The remaining factors were more similar after the fourth factor (ranging from 1.38 for the fifth factor to 1.10 for the tenth factor). Three major dietary patterns were clearly identified for further analysis. The factor scores for each pattern were constructed by summing up the observed intake of the component food items, weighted by the individual factor loadings. The factor scores rank individuals according to their agreement with each dietary pattern. The healthiest dietary pattern was selected, that is, the Healthy Dietary Pattern (principal components analysis), because a high factor score is associated with the healthiest pattern, which is also the case for the Healthy Eating Index and the Mediterranean Diet Score. This Healthy Dietary Pattern was associated with a high intake of fruits, vegetables, nuts, fish, whole grain and low-fat dairy products.

For descriptive statistics, means and standard deviations were calculated for the individual characteristics, according to beer and wine consumption, using chi-square tests for categorical data and unpaired *t*-tests for continuous data.

Participant characteristics included age (20 to 29 years, 30 to 39 years, 40 to 49 years, 50 to 59 years); Body Mass Index (BMI) according to the World Health Organization classifications as normal weight, 18.5 ≤ BMI < 25.0 kg/m2, overweight, 25.0 ≤ BMI < 30.0 kg/m2 and obesity, BMI ≥ 30.0 kg/m2; physical activity levels (stratified as low, moderate and high according to the IPAQ); actual smoking (yes or no); and beer and wine consumption. Despite the non-normal distribution of the scores, means and standard deviations are presented for simplicity. Non-parametric statistical tests were also performed, although the results did not differ from the parametric tests. A two-sided 0.05 level of significance was defined. IBM SPSS Statistics for Windows Version 20.0 (Armonk, NY: IBM Corp) software was used.

Three logistic regression models were constructed with beer versus wine as the dependent variables and with age, military rank (coded as dummy variables for non-commissioned officers versus soldiers and officers versus soldiers), body mass index (coded as dummy variables for overweight versus normal and obese versus normal), physical activity (coded as dummy variables for moderate versus low and high versus low), actual smoking, total energy intake and dietary pattern as independent variables.

## Results

Table 1 presents the demographic and lifestyle characteristics of the participants. Out of the 5000 selected men, only 34 % participated in the study, i.e., responded to the three questionnaires. The most prevalent age category was 40–49 years, and 77 % of participants were non-smokers. Approximately 60 % had a BMI ≥ 25.0 kg/m^2^, while 12.5 % were officers, 51.5 % were non-commissioned officers, and 36 % were soldiers. Responders to the mailing tended to be older than non-responders (*P* < 0.05), and soldiers were less inclined to participate in the study than officers and non-commissioned officers (*P* < 0.05). Approximately 62 % of participants were beer consumers, and 26 % were wine consumers.

**Table 1:** Characteristics of the study participants [*n* (%)]

Characteristics		Responders (*n* = 1699)	Non-responders (*n* = 3301)
Age (year)	20–29	103 (6.1)	477 (14.5)
	30–39	329 (19.4)	782 (23.7)
	40–49	979 (57.6)	1524 (46.2)
	50–59	288 (17.0)	518 (15.7)*
Military rank	Officers	212 (12.5)	386 (11.7)
	Non-commissioned officers	875 (51.5)	1228 (37.2)
	Soldiers	612 (36.0)	1687 (51.1)*
BMI (kg/m^2^)^a^	Normal (<25.0)	685 (40.3)	
	Overweight (≥25.0 to <30.0)	786 (46.3)	
	Obesity (≥30.0)	228 (13.4)	
PA^a^	Low	354 (20.8)	
	Moderate	372 (21.9)	
	High	973 (57.3)	
Actual smoking		394 (23.2)	
Alcohol consumption	Beer^b^	1054 (62.0)	
	Wine^b^	272 (16.0)	
	Others^b^	373 (22.0)	

*P < 0.05

^a^ *BMI* body mass index, *PA* physical activity; BMI was classified according to the World Health Organization [19]; PA was stratified as low, moderate and high according to the International Physical Activity Questionnaire [20]

^b^Beer consumers were defined as those for whom 60 % or more of their total alcohol consumption was beer; wine consumers were defined as those for whom 60 % or more of their total alcohol consumption was wine

Tables 2 and 3 present the factor groupings used in the principal component analysis and the factor-loading matrix for the three major factors identified by using food consumption data from the food frequency questionnaire. The greater the factor loading for a specific food or food item, the greater is the effect of that food or food item on a specific factor. The first factor was heavily loaded with red meats, processed meats, beer, garlic, tomatoes, wine, eggs, poultry, liquor, organ meats and vegetables. This factor explained 7.4 % of the total variance. This factor was labeled the meat dietary pattern. The second factor, explaining 7.2 % of the total variance, included more tomatoes, fruit, low-fat dairy products, whole grain, vegetables, cold breakfast cereals, fruit juice, fish, tea and nuts. This was labeled as the healthy dietary pattern. The last factor, explaining 6.2 % of the total variance, was heavily loaded with red meats, processed meats, sweets, desserts, snacks, high-energy drinks, high-fat dairy products, refined grains, mayonnaise and potatoes. This was labeled as the sweet dietary pattern.

**Table 2:** Factor groupings used in the dietary pattern analysis

Food or food group	Food item
Processed meats	Processed meats, bacon, hot dogs, salami, sausage, ham
Red meats	Beef, pork, lamb, hamburger
Organ meats	Liver
Fish	Fish
Poultry	Chicken or turkey
Eggs	Eggs and all types of preparations with eggs
Butter	Butter
Margarine	Margarine
Low-fat dairy products	Skim or low-fat milk or yogurt or chocolate milk, buttermilk, low-fat cheese
High-fat dairy products	Whole milk or yogurt or chocolate milk, half-and-half milk, cream, ice cream, all types of cheese
Liquor	Liquor
Wine	Red and white wine
Beer	Beer
Tea	Tea
Coffee	Coffee
Fruit	Oranges, grapefruit, raisins, grapes, bananas, fresh apples or pears, strawberries, apricots, nectarines, cherries, kiwi, pineapple, peaches, plums
Fruit juice	Orange juice, other fruit juice
Vegetables	Broccoli, coleslaw and uncooked cabbage, cooked cabbage, cauliflower, Brussels sprouts, kale, sauerkraut, carrots, yams, spinach, iceberg or other lettuce, celery, mushroom, green pepper, eggplant, all other vegetables
Legumes	Beans, peas, lentils, soybeans
Tomatoes	Tomatoes, tomato juice
Garlic	Garlic
Potatoes	Potatoes
French fries	French fries
Whole grain	Cooked breakfast cereals, dark bread, brown rice, other grains, wheat germ
Cold breakfast cereals	Cold breakfast cereals
Refined grains	White bread, biscuits, white rice, pasta, sandwiches
Snacks	Potato chips, pancakes
Nuts	Nuts
High-energy drinks	Cola or other beverages with sugar
Low-energy drinks	Cola or other beverages without sugar
Oil, vinegar	Oil, vinegar
Mayonnaise	Mayonnaise, dressings
Soup	Home-made or ready-made soup
Sweets and desserts	Chocolate, candy bars, cookies, cake, pie, pastry, sugar, jam, waffles

**Table 3:** The factor-loading matrix for the major factors identified by the food consumption data from the food frequency questionnaire

Food or food group	Factor 1 (meat dietary pattern)	Factor 2 (healthy dietary pattern)	Factor 3 (sweet dietary pattern)
Red meats	0.60		
Processed meats	0.58		
Beer	0.47		
Garlic	0.43		
Tomatoes	0.43		
Wine	0.40		
Eggs	0.38		
Poultry	0.37		
Liquor	0.37		
Organ meats	0.33		
Fruit		0.58	
Low-fat diary products		0.47	
Whole grain		0.43	
Vegetables		0.39	
Cold breakfast cereals		0.38	
Fruit juice		0.37	
Fish		0.36	
Tea		0.32	
Nuts		0.30	
Sweets and desserts			0.53
Snacks			0.45
High-energy drinks			0.42
High-fat dairy products			0.40
Refined grains			0.36
Mayonnaise			0.30
Potatoes			0.30

Absolute values <0.30 were excluded from the table for simplicity. Foods or food groups with factor loadings of 0.30 for three factors were excluded; see Table 3 for food groupings. The percentage of explained variance was 7.4 % for factor 1, 7.2 % for factor 2 and 6.2 % for factor 3

Table 4 presents the distribution of the baseline characteristics of participants according to beer and wine consumption. In total, 1054 (62 %) participants were beer consumers and 272 (16 %) were wine consumers. Beer consumers were younger and less likely to be officers. Adiposity was not different between the categories. Wine drinkers were physically more active than beer drinkers, with only 13.6 % of wine drinkers considered to have low activity versus 22.6 % of beer drinkers. Almost 26 % of beer drinkers were smokers, compared with only 16 % of wine drinkers. Beer consumption was associated with higher energy consumption and total and saturated fat intake but less added sugar consumption, which composed only 16.8 energy-percent versus 18.4 energy-percent for wine drinkers.

**Table 4:** Baseline characteristics according to beer and wine consumption (*n* = 1326)

Item	Beer consumers^b^ (*n* = 1054)	Wine consumers^b^ (*n* = 272)	*P*-value*
Age (year)	42.5 ± 7.2	44.8 ± 5.9	<0.001
Military rank [*n* (%)]			0.002
Officers	114 (10.8)	48 (17.6)	
NCO	551 (52.3)	145 (53.3)	
Soldiers	389 (36.9)	79 (29.0)	
BMI^a^ (kg/m^2^) [*n* (%)]			0.360
Normal (<25.0)	421 (39.9)	121 (44.5)	
Overweight (≥25.0 to <30.0)	493 (46.8)	115 (42.3)	
Obesity (≥30.0)	140 (13.3)	36 (13.2)	
PA [*n* (%)]			0.003
Low	238 (22.6)	37 (13.6)	
Moderate	244 (23.1)	63 (23.2)	
High	572 (54.3)	172 (63.2)	
Smoking [*n* (%)]	272 (25.8)	44 (16.2)	0.001
Beer (ml/d)	321 ± 361	27 ± 37	<0.001
Wine (ml/d)	39 ± 58	104 ± 111	<0.001
Total energy (kcal/d)	3128 ± 897	2894 ± 896	<0.001
Protein (energy-percent)	16.3 ± 3.1	17.0 ± 3.5	0.001
Total fat (energy-percent)	37.5 ± 7.7	35.2 ± 8.1	<0.001
Saturated fat (energy-percent)	14.5 ± 4.1	13.5 ± 4.2	<0.001
Total carbohydrates (energy-percent)	41.7 ± 7.4	44.3 ± 8.4	<0.001
Added sugar (energy-percent)	16.8 ± 5.9	18.4 ± 6.6	<0.001
Alcohol (energy-percent)	1.1 ± 0.8	0.8 ± 0.6	<0.001
Sodium (mg/d)	3534 ± 1409	3329 ± 1284	0.030
Calcium (mg/d)	1134 ± 620	1166 ± 661	0.463
Iron (mg/d)	21.9 ± 10.7	21.8 ± 9.8	0.899
Vitamin C (mg/d)	199 ± 127	225 ± 162	0.005
HEI _2010 (range 0 to 100)	54.6 ± 10.4	57.8 ± 10.1	<0.001
MDS (range 0 to 9)	4.0 ± 1.7	4.5 ± 1.8	0.001
HDP (PCA) (range −3.6 to 4.8)	−0.13 ± 0.94	0.38 ± 1.02	<0.001

*P after chi-square for categorical data and unpaired *t*-test for continuous data

*BMI* body mass index, *NCO* non-commissioned officers, *PA* physical activity, *HEI*_*2010* Healthy eating index 2010, *MDS* Mediterranean diet score, *HDP (PCA)* healthy dietary pattern (principal component analysis)

^a^BMI was classified according to the World Health Organization [19]; PA was stratified as low, moderate and high according to the International Physical Activity Questionnaire [20]

^b^Beer consumers were defined as those for whom 60 % or more of their total alcohol consumption was beer; wine consumers were defined as those for whom 60 % or more of their total alcohol consumption was wine

Regarding the dietary patterns, beer consumers scored lower on the healthy eating index 2010, the Mediterranean diet score and the healthy dietary pattern (principal component analysis).

Table 5 presents the results of the logistic regressions with beer versus wine consumption as the dependent variable and socioeconomic and lifestyle variables as the independent variables. The three models differed by the type of dietary pattern score included. For the three models, beer consumption decreased with increasing age, military rank, physical activity and dietary pattern scores. Beer consumption increased with total energy intake and among smokers, except for those adhering to the healthy dietary pattern (principal component analysis) model. BMI did not differ among the models.

**Table 5:** Logistic regressions with beer versus wine consumption as the dependent variable

Dependent variable	Independent variable	b	Se	Odds ratio	95 % CI	*P*-value
Beer vs wine^b^	Constant	5.23	0.76			<0.001
	Age (years)	−0.06	0.01	0.94	0.92–0.96	<0.001
	NCO vs soldiers	−0.06	0.16	0.94	0.69–1.29	0.707
	Officers vs soldiers	−0.62	0.23	0.54	0.35–0.84	0.006
	BMI^a^ overweight vs normal	0.26	0.15	1.30	0.96–1.75	0.089
	BMI^a^ obese vs normal	0.35	0.23	1.43	0.91–2.23	0.122
	PA moderate vs low	−0.46	0.23	0.63	0.40–0.99	0.049
	PA high vs low	−0.78	0.21	0.46	0.31–0.69	<0.001
	Actual smoking	0.39	0.19	1.47	1.02–2.13	0.038
	Total energy (for 100 kcal)	0.03	0.01	1.03	1.01–1.05	0.001
	HEI_2010	−0.03	0.01	0.97	0.96–0.99	<0.001
Beer vs wine^b^	Constant	3.96	0.64			<0.001
	Age (year)	−0.06	0.01	0.94	0.92	<0.001
	NCO vs soldiers	−0.07	0.16	0.93	0.98	0.662
	Officers vs soldiers	−0.60	0.23	0.55	0.35	0.008
	BMI^a^ overweight vs normal	0.26	0.15	1.29	0.96	0.091
	BMI^a^ obese vs normal	0.27	0.29	1.30	0.83	0.244
	PA moderate vs low	−0.41	0.23	0.66	0.42	0.074
	PA high vs low	−0.75	0.20	0.47	0.32	<0.001
	Actual smoking	0.40	0.19	1.49	1.03	0.033
	Total energy (for 100 kcal)	0.03	0.01	1.03	1.01	0.001
	MDS	−0.10	0.04	0.90	0.83	0.015
Beer vs wine^b^	Constant	2.96	0.66			<0.001
	Age (year)	−0.06	0.01	0.94	0.92	<0.001
	NCO vs soldiers	−0.07	0.17	0.93	0.68	0.678
	Officers vs soldiers	−0.60	0.23	0.55	0.35	0.009
	BMI^a^ overweight vs normal	0.28	0.16	1.32	0.97	0.074
	BMI^a^ obese vs normal	0.34	0.23	1.41	0.89	0.139
	PA moderate vs low	−0.37	0.23	0.69	0.44	0.120
	PA high vs low	−0.59	0.21	0.56	0.37	0.005
	Actual smoking	0.22	0.19	1.24	0.85	0.255
	Total energy (for 100 kcal)	0.05	0.01	1.05	1.03	<0.001
	HDP (PCA)	−0.63	0.08	0.54	0.46	<0.001

*BMI* body mass index, *NCO* non-commissioned officers, *PA* physical activity, *HEI*_*2010* Healthy eating index 2010, *MDS* Mediterranean diet score, *HDP (PCA)* healthy dietary pattern (principal component analysis)

^a^BMI was classified according to the World Health Organization [19]; PA was stratified as low, moderate and high according to the International Physical Activity Questionnaire [20]

^b^Beer consumers were defined as those for whom 60 % or more of their total alcohol consumption was beer; wine consumers were defined as those for whom 60 % or more of their total alcohol consumption was wine

## Discussion

In the present study with a military population, daily beer consumption compared with wine is associated with a less healthy dietary pattern, as measured by three different methods, i.e., the healthy eating index 2010, the Mediterranean diet score and a pattern obtained by principal component analysis, and with less physical activity and a higher prevalence of smoking. This means that daily wine consumption is associated with a cluster of healthier lifestyle characteristics, which could influence the relationship between the type of alcoholic beverage consumed and mortality.

Our results are in agreement with other studies carried out in Denmark, the United States, and France [10–12]. Johansen et al. [10] found that Danish people who bought wine purchased a greater number of healthy food items than those who bought beer. Wine buyers bought more Mediterranean food items, and beer buyers bought more traditional Danish food items. Tjønneland et al. [11] found that wine intake was associated with a higher intake of fruit, fish, cooked vegetables, salad, and olive oil. In the same study, wine intake was associated with not smoking and higher education. In a French study, Ruidavets et al. [12] found that wine consumption was also associated with a healthier diet, not smoking and a higher socioeconomic status.

A healthier lifestyle characterized by a higher intake of plant foods, lower intake of saturated fatty acids, and non-smoking has been shown to reduce the risk of premature mortality [13]. The differential relationship between wine and beer consumption and mortality detected by Renaud et al. [1] could be confounded by a healthier lifestyle associated with wine consumption. Studies relating wine to morbidity and mortality do not always fully adjust for dietary patterns. For example, a French prospective cohort study found that moderate wine consumption was associated with a lower risk of mortality compared with beer consumption [14]. The relative risk of mortality was adjusted for age, education, smoking, physical activity, serum cholesterol and BMI but not for dietary patterns, which could explain the observed differential health effects between beer and wine [14].

The two hypothesis-oriented approaches, i.e., the healthy eating index 2010 and the Mediterranean Diet Score, have a different approach to determining alcohol consumption. In the healthy eating index 2010, calories from alcohol are considered to be empty calories, i.e., counted in the empty calorie component, but only when alcohol is consumed beyond moderate amounts. The least restrictive of the two levels defined as moderate drinking in the American Dietary Guidelines (two drinks per day; converted to 28 g of ethanol), was used to set the threshold for counting alcohol as empty calories. This method differs from that of the Mediterranean diet score in which alcoholic beverages result in one point when consuming alcoholic beverages in moderation, which represents 10 % of the total score. Abstainers and consumers of more than three glasses of alcoholic beverages per day are classified in the same categories with a score of zero for the alcoholic beverages component. It is important to note that this classification of alcoholic beverages in the dietary indices occurs independently of the type of alcohol consumed. The total explained variance by principal component analysis for the three dietary patterns in our study was only 21 %. However, this low percentage is in agreement with Slattery et al. [15] and Schulze et al. [16], who found that dietary patterns accounted for 23 and 30 % of the variance, respectively.

A limitation of this study is the low response rate of 34 %. However, the main purpose of this study was not to provide exact estimations of prevalence but to detect differences in dietary patterns between participants based on the type of alcoholic beverages consumed. In their publication, Lorant et al. [17] found that less educated subjects were less likely to participate in a survey when they had a poor health status compared with better-off groups. This may lead to an underestimation of the relationship between alcoholic beverage consumption and dietary patterns. Another limitation of this study is the use of a food frequency questionnaire to register alcoholic beverage consumption. Working with predefined categories of consumption can limit choices for the participants, resulting in lower sensitivity. In 2004, a national food consumption survey was carried out in Belgium among a representative group [18]. The mean (SD) beer and wine consumption for men aged 19 to 59 years was 236 ml (297) and 84 ml (109), respectively. In this military population, beer and wine consumptions was 222 ml (338) and 51 ml (75) per day, respectively. This study had a cross-sectional design in which exposure and outcomes were determined simultaneously. Such a design is efficient for hypotheses generation, but because of the lack of a temporal relationship between the exposure and outcome, causal analyses are impossible.

## Conclusion

In conclusion, in this military population, wine consumption was associated with a healthier lifestyle than beer consumption. Those differences must be taken into account when relating alcoholic beverage consumption with health-related outcomes.
